# PREDICTING ALL-CAUSE MORTALITY USING THE SARCOPENIA PHENOTYPE: EVIDENCE FROM THE KFACS

**DOI:** 10.1093/geroni/igae098.4048

**Published:** 2024-12-31

**Authors:** Miji Kim, Hyung Eun Shin, Daehyun Lee, Heeeun Jung, Jae Young Jang, Hyunjin Cho, Nahyun Lim, Chang Won Won

**Affiliations:** Kyung Hee University, Seoul, Republic of Korea; Kyung Hee University, Seoul, Republic of Korea; Kyung Hee University, Seoul, Republic of Korea; Kyung Hee University, Seoul, Republic of Korea; Kyung Hee University, Seoul, Republic of Korea; Kyung Hee University, Seoul, Republic of Korea; Kyung Hee University, Seoul, Republic of Korea; Kyung Hee University, Seoul, Republic of Korea

## Abstract

The Global Leadership Initiative on Sarcopenia (GLIS) has sought to establish a consensus on sarcopenia diagnosis, which involves reduced muscle mass and strength. We investigated the association between sarcopenia phenotypes (low muscle mass, muscle strength, and physical performance) and all-cause mortality. We conducted a prospective 6-year follow-up analysis of 2,400 older adults (52.8% women; mean age 76.0±3.9 years) enrolled in the Korean Frailty and Aging Cohort Study. Participants were included if they had undergone dual-energy X-ray absorptiometry, handgrip strength testing, and physical performance assessments at baseline (2016–2017). Discrete-time Cox proportional hazards models evaluated the association between sarcopenia and all-cause mortality. According to the GLIS criteria, the prevalence of sarcopenia was 15.4% in men and 7.5% in women. Totally, 203 deaths occurred, with 6-year mortality rates of 2.1 (95% confidence interval [CI]: 1.9–2.5) per 100 person-years in individuals without sarcopenia, and 4.8 per 100 person-years (95% CI: 3.6–6.3) in those with sarcopenia. After adjusting for potential confounders, reduced muscle mass and muscle strength were significantly associated with an increased risk of all-cause mortality (hazard ratio [HR]: 1.56, 95% CI: 1.09–2.24). Moreover, the presence of all three criteria of sarcopenia was associated with an increased risk of all-cause mortality (HR: 1.87, 95% CI: 1.27–2.67), while reduced muscle mass and physical performance were not significantly associated with mortality (p=0.06). In conclusion, the conceptual definition of sarcopenia outlined by the GLIS, which includes both reduced muscle mass and muscle strength, is associated with premature mortality among community-dwelling older adults.

